# Editorial: Planetary health challenges: interventions for effective knowledge mobilization for policy- and decision-makers and science communication

**DOI:** 10.3389/fpubh.2025.1738710

**Published:** 2025-12-04

**Authors:** Enrique Castro-Sánchez, Priscila de Morais Sato, Miquel Bennasar-Veny

**Affiliations:** 1 National Institute for Health and Care Research (NIHR) Health Protection Research Unit in Healthcare-Associated Infection and Antimicrobial Resistance, Imperial College London, London, United Kingdom; 2 UK Health Security Agency, London, United Kingdom; 3 Global Health Research Group, Universitat de les Illes Balears, Palma, Spain; 4 Federal University of Bahia, Salvador, Brazil; 5 Nursing and Physiotherapy Department, University of the Balearic Islands, Palma, Spain; 6 Research Group on Nursing, Community and Global Health, Health Research Institute of the Balearic Islands (IdISBa), Palma, Spain; 7 Centre for Biomedical Research Network in Epidemiology and Public Health Consorcio de Investigación Biomédica en Red de Epidemiología y Salud Pública (CIBERESP), Madrid, Spain

**Keywords:** planetary health, knowledge mobilization, equity, low-and- middle-income countries, framework

The collection of papers within this Research Topic “*Planetary Health Challenges: Interventions for Effective Knowledge Mobilization for Policy- and Decision-Makers and Science Communication*” arrives at a moment of profound urgency. Planetary health (PH), the interdependency of human civilization and the natural systems that support it, is defined by “wicked problems” like the climate emergency, drug resistance, and socioeconomic injustice (1). These problems are complex, defying simple, siloed solutions. The core challenge is no longer merely identifying these threats, but rather bridging the ever-increasing gap between scientific knowledge and meaningful policy and societal action (2). As highlighted by Mulopo et al., this gap persists particularly in low- and middle-income countries (LMICs), where knowledge translation research remains underrepresented in international literature, raising equity concerns.

This Research Topic provides foundational analysis, novel frameworks, and critical insights into this “know-do” gap, focusing intently on the mechanisms required to integrate evidence across the research, policy, and citizen sectors. By scrutinizing knowledge translation efforts, developing tools for inclusive stakeholder engagement, and analyzing the barriers to behavioral change, these contributions collectively chart a more pragmatic and equitable path toward addressing humanity's greatest existential threats.

## Bridging the “know-do” gap: fragmentation and urgency

The foundational challenge of planetary health lies in the knowledge mobilization (KM) and knowledge translation (KT) processes. As Mulopo et al. articulate in their systematic scoping review on the translation of climate change research into public health action, KT refers to the targeted efforts to make research evidence actionable for decision-makers. The authors, however, expose a critical disparity: a significant lack of primary studies on KT and climate change, particularly in LMICs. This is a profound equity concern, as LMICs are simultaneously the least responsible for global emissions and the most vulnerable to climate-related health impacts (3) yet they remain underrepresented in the research that should inform their adaptive strategies.

Mulopo et al.'s findings emphasize the need to move beyond traditional dissemination to approaches that foreground citizen engagement, co-production, and advocacy to strengthen health system resilience. Their categorization of successful KT approaches, such as monitoring media coverage, establishing communities of practice, and leveraging health impact assessment tools, provides a robust framework, albeit one currently rooted in high-income country experiences.

Complementing this, Menezes et al. address the structural barriers inherent in the PH field itself. In their opinion piece, they highlight that the very interdisciplinary nature of planetary health leads to fragmented efforts, often with non-interoperable data, distinct terminologies, and methodologies that result in scientific expertise remaining in isolated “bubbles.” To overcome this, they advocate for a “pragmatic approach” that emphasizes targeted, actionable goals. This involves strengthening data integration platforms across sectors (like environmental, public health, and social sciences), standardizing language, and integrating research initiatives with governmental agencies from the outset. Their focus on Brazil exemplifies how comprehensive national registries are useless without the political will and technological infrastructure to connect and leverage them for policy implementation (4).

## The nexus of policy and citizen engagement

Effective knowledge mobilization is not a one-way street from scientists to policymakers, but a complex, reciprocal process involving diverse stakeholders and recognizing lived experience.

The complexity of the policy and decision-making environment is precisely what Black and Bates address in their detailed study on stakeholder analysis in urban-planetary health research. The sheer scale and complexity of urban systems, where decisions on housing, transport, and land use profoundly affect health, demands a methodical approach to engagement. They introduce the “Key Group Approach,” a practical, iterative method to identify and analyse stakeholders based not just on their influence and interest, but on their specific knowledge domains. This framework is essential for ensuring a sample of participants is genuinely representative, helping researchers identify critical gaps in expertise, such as law, finance, or infrastructure planning. Crucially, their method also forces consideration of groups with little or no voice, such as future generations or the natural world itself (non-human “stakeholders”), demanding researchers account for these hidden attributes in their findings.

The perspective is further deepened by Delbaere et al., who focus on the citizen's lived experience. They propose the Environmental Health Citizen Interview Tool as a qualitative method to capture diverse perspectives on environmental wellbeing, explicitly grounded in the principles of epistemic justice and inclusion. By moving beyond traditional quantitative measures, their tool ensures that conceptualizations of wellbeing are not limited to the findings of WEIRD (“Western, Educated, Industrialized, Rich, Democratic”) societies. The tool operationalizes environmental wellbeing by inquiring about subjective experiences related to air quality, noise, green/blue spaces, and institutional and social determinants. This inclusion of diverse voices, particularly those often marginalized (e.g., ethnic minorities, LGBTQI+ populations), is critical. Their work is a reminder that successful planetary health interventions, such as Nature-based Solutions (NbS), must be co-designed to ensure benefits are equitably distributed and culturally sensitive, rather than imposed top-down (5).

## Transforming practice: the case of sustainable healthcare

The challenge of translating knowledge into action is powerfully illustrated in the healthcare sector, which paradoxically contributes nearly 5% of global carbon emissions (6). Scholz et al. conducted a survey in Germany to gauge patient perspectives on climate action in hospitals. Their findings expose a classic attitude-behavior gap: while patients express high environmental awareness in their personal lives and strong support for sustainable healthcare practices (like greener procurement and reduced length of stay), this enthusiasm wanes when asked to accept personal financial costs (e.g., higher insurance premiums) or discomforts (e.g., giving up meat dishes).

This research highlights that while patient education (using tools like a “Nutri-Score” for treatment carbon footprints) is valuable for raising awareness, it is unlikely to be a primary driver of systemic change. Instead, the authors conclude that patients predominantly see the state's obligation to establish legally binding frameworks and policy changes. This underscores a crucial lesson for knowledge mobilization: for high-cost, high-impact sectors like healthcare, the greatest potential lies not in burdening the individual citizen but in advocating for regulatory and market-based interventions that make sustainable choices the default, maintaining quality of care as a non-negotiable standard.

## Conclusion

The five papers in this Research Topic reinforce a cohesive message: the successful transition toward planetary health relies on sophisticated, integrated, and equitable knowledge mobilization. They move the conversation beyond problem definition toward offering concrete frameworks (Black and Bates), operational tools (Delbaere et al.), systematic analysis (Mulopo et al.), and critical policy feedback (Menezes et al.; Scholz et al.).

Ultimately, PH depends on our collective capacity to mobilize and translate knowledge not only across disciplines, but across boundaries of geography, equity, and governance. The collective evidence demands a recalibration of our approach to the science-policy interface: we must adopt pragmatic, data-driven strategies to break down disciplinary silos, invest in inclusive co-design to honor epistemic justice, and focus advocacy efforts on systemic regulatory change rather than individual behavioral burdens. The urgent next step is to test and adapt these frameworks and tools within the currently underrepresented research of LMICs contexts, ensuring that all regions, particularly the most affected, can effectively translate planetary health knowledge into resilient, sustainable futures.
